# Current trials for frontline therapy of mantle cell lymphoma

**DOI:** 10.1186/s13045-018-0556-x

**Published:** 2018-01-27

**Authors:** Raphael E. Steiner, Jorge Romaguera, Michael Wang

**Affiliations:** Department of Lymphoma and Myeloma, The University of Texas MD Anderson Cancer Center, 1515 Holcombe Boulevard, Houston, TX 77030 USA

**Keywords:** Mantle cell lymphoma, Frontline treatment, Clinical trials, Novel agents

## Abstract

Mantle cell lymphoma (MCL) is a rare and incurable subtype of non-Hodgkin’s lymphoma that generally affects older individuals. However, the use of high-dose therapy and autologous stem cell transplant has improved significantly the prognosis of this hematological malignancy, but at the cost of increased toxicities, such as acute toxic death and secondary malignancies. But thanks to a rising understanding of the biology of MCL, the explosion of specifically targeted new efficacious agents, immunotherapy agents, and cellular therapies in the frontline setting, the prognosis of MCL is expected to improve dramatically.

The initial treatment of MCL is currently not standardized and the therapeutic landscape of MCL is rapidly evolving. This review provides an extensive overview of the current frontline therapy trials for MCL and presents the results of innovative regimen, including some integrating novel agents and desintensified chemotherapy.

## Background

Mantle cell lymphoma (MCL) has been recognized as an aggressive but incurable small B cell lymphoma and represents only 2.8% of non-Hodgkin lymphoma (NHL) in the USA [1, 2]. MCL has an overall increasing incidence in the USA and incidence rate in men (0.84) is more than twice that of women (0.34). The median age at diagnosis is 68 years [1].

The 2016 revision of the World Health Organization (WHO) classification of lymphoid neoplasms recognizes classical mantle cell lymphoma and two types of clinically indolent variants, leukemic non-nodal MCL, and in situ mantle cell neoplasia (ISMCN) [3].

Classical MCL (cMCL) is generally composed of immunoglobulin heavy chain variable region (IGHV) unmutated or minimally mutated B cells that frequently express SOX11. cMCL typically involves lymph nodes and other extra-nodal sites. Acquisition of additional molecular/cytogenetic abnormalities such as TP53 can lead to even more aggressive blastoid or pleomorphic MCL [3–5]. It is to be noted that cyclin D1 (CCND1) protein overexpression and/or the t(11;14)(q13;q32) translocation are the pathognomonic hallmarks of MCL. Nevertheless, SOX11 expression can be used as a biomarker for most cases of MCL that lacked both t(11;14) and cyclin D1 protein but still had a gene expression profile suggesting a diagnosis of MCL [6]. Another subtype of MCL develops from IGHV-hypermutated, SOX11 negative B cells. This subtype leads to leukemic non-nodal MCL, usually involving the peripheral blood, bone marrow, and often spleen. These cases are frequently clinically indolent; however, secondary abnormalities, often involving TP53, may occur and lead to very aggressive disease.

Lastly, ISMCN, which is cyclin D1 positive and involves the inner mantle zones of follicles has a low rate of progression. ISMCN is indolent and can be observed until progression [7].

Most patients will require a combination of chemotherapy plus immunotherapy (i.e., chemoimmunotherapy) at the time of diagnosis in order to improve outcome in the aggressive variants of MCL [8]. But thanks to intensive frontline therapies, young patients (≤ 65 years) can reach the median survival of 12.7 years with the NORDIC regimen (rituximab and augmented CHOP (cyclophosphamide, doxorubicin, vincristine, prednisolone) regimen (maxi-CHOP) alternating with high-dose cytarabine (AraC)) followed by BEAM (carmustine, etoposide, Ara-C, melphalan) or BEAC (carmustine, etoposide, Ara-C, cyclophosphamide) used as a high-dose regimen before autologous stem cell transplant (ASCT) [9]. Furthermore, young patients (≤ 65 years) can even reach a median overall survival (OS) of 13.4 years when treated with rituximab plus hyper-CVAD alternating with MTX/Ara-C (rituximab plus fractionated cyclophosphamide, vincristine, doxorubicin, and dexamethasone alternating with rituximab plus high-dose methotrexate-cytarabine) [10]. However, these intensive frontline therapies are a double-edged sword considering acute toxic deaths and the rate of secondary malignancies (solid tumors 9.4% [9], myelodysplastic syndromes (MDS)/leukemia 3.1–6.2% [9, 10]). Besides, stem cell transplants are available only at major hospitals and rituximab plus hyper-CVAD alternating with MTX/Ara-C is too toxic to be done in the majority of community hospitals [11].

In contrast, the choice of treatment of untreated MCL (UMCL) for frail patients or patients aged ≥ 65 years represents a trade-off between life extension and limitation of toxicity.

Initial treatment of MCL is not standardized and with new therapies, notably immune therapies and targeted therapies (Table 1) combined or not with cytotoxic therapy, the therapeutic landscape of MCL is rapidly evolving [12]. This review provides an extensive overview of the current frontline therapy trials for MCL, in order to evaluate putative beneficial roles of novel therapy agents and their toxicities.

**Table 1:** Mechanisms of novel drugs for UMCL used in the trials described

BTK inhibitor	Ibrutinib
	Acalabrutinib
	Bgb-3111
Anti-CD20	Rituximab
	Obinutuzumab
	Ofatumumab
	Tositumomab
	Anti-B1 antibody
	Ibritumomab
Alkylating agent	Bendamustine
Immuno-modulator	Thalidomide
	Lenalidomide
Purine analog	Cladribine
Proteasome inhibitor	Bortezomib
mTOR inhibitor	Temsirolimus
Radioisotope	Iodine ^131^I Tositumomab
	^131^I Anti-B1 antibody
	^90^Y-Ibritumomab tiuxetan

*BTK* Bruton’s tyrosine kinase, *CDK* cyclin-dependent kinase, *mTOR* mechanistic target of rapamycin

We present in this review the different trials and their outcome (if available) sorted according to age groups at diagnosis (older, ≥ 65 years old; young, 18 years to 65 years old; all ages, 18 years and older). It is to be noted that most studies use the cutoff of 65 years to differentiate younger and older population; however, this cutoff is not universal. Hence, to simplify the repartition of clinical trials, we tolerated some overlap between the groups by drawing the cutoff of 65 years with a margin of ± 5 years. This article will include ongoing trials, trials in in the recruitment stage, and completed trials, but will not include published trials.

## Methods, search strategies, and selection criteria

We searched for all the trials posted on clinicaltrials.gov from January 2000 until January 6, 2018, for the terms *mantle cell untreated*, *mantle cell frontline*, *mantle cell front line*, *mantle cell newly diagnosed*, and *mantle cell new diagnosis.* Results available on clinicaltrials.gov and pubmed.gov and results available on abstracts from annual meetings of the American Society of Hematology (ASH) are presented in this review. However, trials not registered on clinicaltrials.gov, trials including other malignancies than MCL, trials including treated mantle cell lymphoma patients only, and trials using rituximab-chemotherapy only were excluded from this review.

## Age and choice of treatment

MCL is a disease affecting mostly elderly people. As shown by the numbers of an analysis with data from 1992 to 2004 by the Surveillance, Epidemiology and End Results data submission, the relative risk of being diagnosed with MCL is 1.00 for patients under 50 years, 11.72 for patients aged 50–59 years, 27.55 for patients aged 60–69 years, 41.70 for patients aged 70–79 years, and 39.10 for patients aged over 80 years old [1]. The choice of therapy for newly diagnosed MCL depends on different factors, such as age, comorbidities, performance status (PS), patient preferences, side effect profiles, and the physician’s comfort with the various regimens, and age alone should not be the sole deciding factor of therapy.

Indeed, a 70-year-old patient with little comorbidities and a good performance status could possibly tolerate a chemoimmunotherapy/ASCT as well as a 60 years old patient with a poor PS and many comorbidities [13, 14]. In order to better integrate these previously mentioned parameters and decide the appropriate intensity of therapy, several geriatric scores have been created, such as the G8 questionnaire (eight items taken in account: food intake, weight loss, mobility, neuropsychological problems, body mass index (BMI), polymedication, perception of owns health, age) and the Flemish version of the Triage Risk Screening Tool (fTRST) (five items taken in account: cognitive impairment, living without assistance, mobility, recent hospitalization, polypharmacy) [15]. In addition, a proper geriatric evaluation is especially helpful for the choice of therapy of patients in the *gray zone*, between 65 and 70 years old, who might require an intermediate approach.

## Chimeric antigen receptor (CAR)-T cell therapy

Chimeric antigen receptor (CAR)-T cells have demonstrated activity in relapsed/refractory MCL (RRMCL) [16–20], but their future in the frontline setting is still relatively unknown. Indeed, all the current CAR-T cell trials for MCL are for RRMCL.

Theoretically, MCL is the ideal neoplasia for the implementation of CAR-T cell therapies due to its incurability and remissions of sufficient depth and duration to allow for manufacturing and administration of CAR-T cells.

In order for CAR-T cell therapies to be more widely adopted in chemorefractory RRMCL, either they will have to demonstrate an ability to safely induce responses in patients who would not be eligible for allogeneic stem cell transplantation due to comorbid conditions or rapidly progressing disease or they will need to be associated with a relatively superior efficacy and tolerability profile. Moreover, the toxicities associated with CAR-T cells at present, including neurologic issues related to cytokine release syndrome [21], may limit applicability to MCL patients who are commonly older and have comorbid conditions [22].

## Trials for older patients

The definition of older patients is necessarily arbitrary and is constantly evolving, partly due to the progress in treating elderly patients with more intense therapies.

While the majority of patients diagnosed with MCL are older than 60 years, most studies of high-dose therapy and autologous stem cell transplant (ASCT) or intensive therapy for MCL have been performed on patients younger than 60 years. Therefore, safety and efficacy for such therapies are not well established in this age group [23]. Indeed, the “choice” of drawing a cutoff of 65 or 60 years to define older in the treatment of MCL has been largely determined by the fact that ASCT became an important frontline treatment modality in MCL because of its proven improvement of progression-free survival (PFS) and OS and the studies that showed this were using old standards of age cutoff to decide eligibility to do consolidation ASCT. However, this arbitrary age cutoff does not take PS and comorbidities into consideration, as explained in the previous section. Consequently, some patients older than 65 years may have been undertreated, which could explain why the survival of this elderly population is clearly inferior to that of younger patients.

Indeed, according to a population-based study from the Swedish Lymphoma Registry, the estimated 3-year survival is of 44% for patients ≥ 60 years and of 81% for patients aged ≤ 60 years. Consequently, there is an urgent need to improve the outcome of this older population [24].

Patients > 65 years old who do not qualify or do not want to participate in a clinical trial are usually recommended against an intensive chemotherapy such as R-HyperCVAD/R/MTX/Ara-C or high-dose chemotherapy and ASCT. In fact, in a phase II trial with 97 patients with UMCL and treated with frontline R-HyperCVAD/R/MTX/Ara-C, the subgroup of 32 patients aged > 65 years presented an inferior failure-free survival (FFS) (3-year FFS 75% for patients aged ≤ 65 years and 3-year FFS 50% for patients aged > 65 years old) and increased dose reduction due to toxicity (in 17% of patients aged ≤ 65 years and in 72% of patients > 65 years old) [25]. This older population needs as well to achieve a durable remission. But with the conventional current treatments available, the choice of therapy is unfortunately a trade-off between duration of survival and toxicity.

In this regard, Kahl et al. proposed a modified hyper-CVAD without methotrexate and cytarabine with rituximab maintenance for previously untreated mantle cell lymphoma. In this phase II trial, 22 patients with UMCL aged 40–81 years (median age 63 years) received this modified regimen and showed an overall response rate (ORR) of 85%, a complete response rate (CRR) of 70%, a 2-year PFS of 73%, and a 2-year OS of 82% [26]. This regimen demonstrates ORR comparable to conventional hyper-CVAD (85 versus 93%), better CRR compared to R-CHOP (70 versus 34–48%), and is less toxic, especially in patients over 60 years.

Later, Kahl et al. added bortezomib to the modified R-HyperCVAD (VcR-CVAD with maintenance rituximab (MR)) in a phase II study with 30 patients with UMCL. With a median age of 61 years, there was an even distribution of patients < 60 years and ≥ 60 years. This regimen showed improved results with an ORR of 90% and a CRR of 77%. The 6-year PFS and OS were 53 and 70% respectively, and there was no difference of 6-year PFS and OS between the age subgroups (age < 60 years and age ≥ 60 years) [27].

Nevertheless, there has been an explosion of specifically targeted new efficacious agents, currently approved in the relapsed setting and studied in the frontline setting. The following paragraph describes the inclusion of these novel therapies in trials designed for patients aged ≥ 60–65 years old (Table 2).

**Table 2:** Clinical trials for older patients with MCL

Leading institution	PI last name	Regimen	Study ID/NCT	EE	Phases	Line	Recruitment	First received	Study results	Cytogenetical inclusion criteria	Age group (years)
MDACC	Wang	RLI	2016-0280 NCT03232307	40	II	1st	Not yet rec	07/2572017	NA	Ki-67 ≥ 50% Cycline D1 +	> 65
Acerta Pharma BV	Gupta	BR vs BR-Acalabrutinib	ACE-LY-308 NCT02972840	546	III	1st	Recruiting	11/21/2016	NA	t(11;14) and/or CCND1 overex	≥ 65
MDACC	Wang	IR	2013-0090 NCT01880567	50	II	1st/RR	Recruiting	6/14/2013	Preliminary [33] but no results for UMCL	If UMCL: Ki-67 < 50% Cycline D1 +	> 65 if UMCL ≥ 18 if RRMCL
MDACC	Wang	BR vs I-BR	SHINE NCT01776840	524	III	1st	Active, not rec	1/24/2013	NA	t(11;14) and/or CCND1 overex	≥ 65
FCCC	Smith	4 arms: A. BR → R B. BRV → R C. BR → LR D. BRV → LR	ECOG-E1411 NCT01415752	332	II	1st	Recruiting	8/11/2011	NA	t(11;14) and/or CCND1 overex	≥ 60
LUH	Jerkeman	L-BR	NLG-MCL4 NCT00963534	60	I/II	1st	Active, not rec	8/20/2009	Preliminary [41] CRR 64% mPFS 42 months 3-year OS 73%	Not specified	≥ 65

*BR* bendamustine rituximab; *CCND1* cyclin D1; *CRR* complete response rate; *EE* estimated enrollment; *FCCC* Fox Chase Cancer Center; *I-BR* ibrutinib bendamustine and rituximab; *ID* identification; *IR* ibrutinib-rituximab; *L* lenalidomide; *L-BR* lenalidomide bendamustine and rituximab; *LR* lenalidomide rituximab; *LUH* Lund University Hospital; *MDACC* MD Anderson Cancer Center; *mPFS* median progression-free survival; *NA* not available; *NCT* clinical trial number registered at clinicaltrials.gov; *OS* overall survival; *overex* overexpression; *PI* principal investigator; *R* rituximab; *rec* recruiting; *RLI* rituximab, lenalidomide, ibrutinib; *RR* relapsed and/or refractory; *t* translocation; *UMCL* untreated mantle cell lymphoma

Ibrutinib is a first-in-class covalent oral inhibitor of Bruton’s tyrosine kinase (BTK), a key component of the B cell receptor (BCR) signaling pathway with a pro-apoptotic effect. Ibrutinib has shown activity as monotherapy or combination therapy for numerous hematological malignancies, including for MCL. In the RRMCL setting, ibrutinib monotherapy showed an ORR 68% [28, 29]. In the frontline setting, ibrutinib has only been studied in two phase I trials including a very small number of patients with UMCL, without statistically significant data regarding efficacy [30, 31].

Furthermore, ibrutinib has been shown to inhibit BCR, chemokine-mediated adhesion, and migration of MCL cells in vitro [32]. This compartmental shift phenomenon led to the hypothesis that when MCL cells are driven out of their micro-environment into the peripheral blood circulation, they are more vulnerable to the targeted attack of rituximab. Consequently, this joined action of ibrutinib and rituximab leads to a stronger antitumor activity [33].

In this regard, MD Anderson Cancer Center (MDACC) sponsors the phase II study 2013-0090 (NCT01880567) with the combination of ibrutinib and rituximab for patients with RRMCL and UMCL. Preliminary results showed for the 50 patients with RRMCL (no results for UMCL patients) aged 45–86 years old (median age 67 years) a 12-month progression-free survival of 75% and overall survival of 85.5% [33]. To be noted, this study is still recruiting and is open as well for UMCL patients > 65 years.

Lenalidomide is an oral derivative of thalidomide drug which has direct activity on malignant B cells and indirect effects mediated through T cell activation and proliferation, enhanced number of natural killer (NK) cells and antibody-dependent cellular cytotoxicity (ADCC), and balance an anti-inflammatory effect on cytokines [34, 35]. Lenalidomide has been studied for different hematological malignancies, including for the treatment of RRMCL [36].

In the phase Ib-II trial, Goy et al. combined rituximab with lenalidomide and ibrutinib (RLI) for RRMCL patients. This study with patients aged 47–81 years (median age 67.5 years) showed very promising preliminary results with CRR of 60%, 6-month PFS of 91%, and 6-month OS of 100% [37].

With these relevant data, MDACC will launch in 2018 the phase II trial 2016-0280 with RLI for UMCL patients aged > 65 years. The primary outcome is the ORR at 4 months of treatment, and this study is expected to be completed in December 2022.

Alternatively, the SHINE trial (NCT01776840) is an international (190 study locations worldwide) phase III two-arm study sponsored by Janssen Research & Development, LLC with patients ≥ 65 years old with UMCL comparing arm A with six cycles of bendamustine rituximab (BR) and placebo versus arm B with six cycles of ibrutinib-BR (I-BR) [30]. The primary outcome is PFS (time frame: up to 7 years after the last patient is randomized); the trial is currently ongoing but not recruiting participants and is expected to be completed in March 2018. There is currently no preliminary result.

To be noted, the use of frontline BR as non-intensive therapy of UMCL for patients aged 18 years and older is validated by a phase III non-inferiority trial comparing BR (261 patients aged 34–83 years, median age 64 years) versus R-CHOP (253 patients aged 31–82 years, median age 63 years) as first-line treatment for patients with indolent lymphomas and mantle cell lymphomas. This trial showed that UMCL patients with BR had a median PFS of 35.4 months for BR versus 22.1 months for patient treated with R-CHOP; OS was not reached in either groups [38]. Patients treated with BR presented an increased PFS and fewer toxic side effects compared to R-CHOP. However, the study did not present a subgroup analysis of UMCL patients aged > 60–65 years old. BR has been proven less toxic than R-CHOP for patients of all ages, and the following trials using the BR backbone required patients to be ≥ 60–65 years to be eligible. Nevertheless, these age criteria do not mean that this studied combination is the best or most appropriate therapy for elderly patients.

Alternatively, acalabrutinib (ACP-196) is an oral selective second-generation BTK inhibitor with improved target specificity over ibrutinib. As a matter of fact, ibrutinib has untoward effects, such as bleeding, rash, and atrial fibrillation, which could be partly due to the bystander effects on targets other than BTK. Consequently, more selective BTK inhibitors (ACP-196, ONO/GS-4059, BGB-3111, CC-292) are being explored [39]. Acalabrutinib has shown impressive activity in the RRMCL setting in the phase II ACE-LY-004 trial with ORR of 81% and CRR of 40%. The 12-month PFS and OS were 67 and 87% respectively. There was no case of atrial fibrillation and one case of grade ≥ 3 hemorrhage [40].

Acerta Pharma BV sponsored a phase III two-arm study with patients ≥ 65 years old with UMCL ACE-LY-308 (NCT02972840). Arm A is with acalabrutinib, rituximab, and bendamustine (ABR); arm B is with placebo and BR. The primary outcome of ACE-LY-308 is PFS per the Lugano Classification for NHL in arm 1 compared to arm 2 (time frame 48 months) and the trial is recruiting. The study is expected to be completed in October 2022, and there is currently no preliminary result.

The international Scandinavian trial of the Nordic Lymphoma Group NLG-MCL4 (NCT00963534) phase I/II with lenalidomide, bendamustine, and rituximab (LENA-BERIT or L-BR) is for patients with UMCL ≥ 65 or < 65 years and unable to tolerate high-dose chemotherapy with autologous stem cell support. This trial is not completed yet but has published the following preliminary results in October 2016. A single arm of 51 patients aged 62–84 years (median age 71 years) received six monthly cycles of L-BR. After six cycles of L-BR, the ORR was 80%, complete remission rate (CRR) was 64%, and 36% were MRD (minimal residual disease) negative. At a median follow-up time of 31 months, the median PFS was 42 months and 3-year overall survival was 73%. However, 42% of patients presented grade 3–5 infections (including three opportunistic infections), 74.5% of patients presented grade 3–4 neutropenia, and 16% of patients presented a secondary primary malignancy (SPM) (chronic myelomonocytic leukemia, 1 Hodgkin lymphoma, 1 renal cancer, 1 squamous epithelial cancer of the skin, 1 squamous epithelial lung cancer in a heavy smoker, 1 hepatocellular carcinoma, and 1 prostate cancer, 2 patients had noninvasive malignancies: 1 with basal cell carcinoma and 1 with squamous cell carcinoma in situ and basal cell carcinoma) [41]. The extent of these adverse events might limit the future use of L-BR. The trial is ongoing but not recruiting participants and was expected to be completed in August 2017.

Bortezomib, a reversible inhibitor of the chymotrypsin-like activity of the 26S proteasome, has been used in combination chemoimmunotherapy for UMCL. The use of bortezomib for UMCL has been validated in the phase III trial LYM-3002 with 487 patients with UMCL aged 26–88 years (median age 66 years old), which compared the outcomes of 244 patients treated with (six to eight cycles of) R-CHOP versus 243 patients treated with (six to eight cycles) of VR-CAP (R-CHOP regimen, but replacing vincristine with bortezomib). The ORR and median PFS were of 89% and 14.4 months in the R-CHOP arm (*p* < 0.001) versus 92% and 24.7 months in the VR-CAP arm and the 4-year OS was of 54 versus 64% respectively. Additional toxic effects were observed in the VR-CAP arm, but without significant increase of the treatment-related mortality. Lastly, unlike currently recommended, rituximab maintenance was not used in this trial, which could have potentially extended the OS [42]. This last trial did not have a subgroup analysis for older patients, so it in unclear if these results apply as well for patients > 60–65 years.

Furthermore, the phase II trial SWOG S0601 with 65 UMCL patients aged 36–85 years old (median age 61 years) treated with VR-CHOP and with bortezomib maintenance for the patients achieving at least a stable disease after induction therapy showed similarly to the previous study an ORR of 80%. The median PFS and 2- and 5-year PFS were 29.5 months and 62 and 28%, and the 2- and 5-year OS were of 85 and 66% respectively. Thus, bortezomib maintenance might extend the disease control [43]. It is to be noted that the SWOG S0601 did not have an age subgroup analysis either.

The international North American phase II study of the Eastern Cooperative Oncology Group with patients ≥ 60 years old with UMCL ECOG-E1411 (NCT01415752) has the four following arms: arm A is with rituximab and bendamustine (BR) followed by rituximab (R) consolidation, arm B is with rituximab, bendamustine, and bortezomib (RBV) followed by rituximab consolidation, arm C is with BR followed by consolidation with lenalidomide and rituximab (LR), and arm D is with RBV followed by consolidation with LR. The primary outcome is 2-year PFS of patients with RBV, and the trial is currently recruiting participants and expected to be completed in March 2019. There is currently no preliminary result.

## Trials for younger patients

For younger patients under 65–70 years without significant comorbidities who do not qualify or do not want to participate in a clinical trial (Table 3), a more intensive approach is recommended with intensive chemoimmunotherapy [44] (such as R-Hyper-CVAD/cytarabine/MTX) or conventional chemoimmunotherapy (such as R-CHOP/R-DHAP (rituximab, cyclophosphamide, doxorubicin, vincristine, and prednisone/rituximab, dexamethasone, high-dose cytarabine, and cisplatin), R-CHOP, or VcR-CAP (bortezomib, rituximab, cyclophosphamide, doxorubicin, and prednisone)) followed by high-dose chemotherapy and ASCT. It is to be noted that until recently, prior trials with ASCT used to include patients until 60–65 years. However, newer trials are nowadays accepting patients up to 70 years and even older [23].

**Table 3:** Clinical trials for younger patients with MCL

Leading institution	PI last name	Regimen	Study ID/NCT	EE	Phase	Line	Recruitment	First received	Study results	Cytogenetical inclusion criteria	Age group (years)
IHBDH	Yi	R-EDOCH/R-DHAP → HDT/ASCR or R-EDOCH/R-DHAP → MR or MTp	IIT2015007-EC-1 BDH-MCL01 NCT02858804	55	IV	1st	Recruiting	7/17/2016	NA	Not specified	18–65
WUSM	Kahl	BR/RAC	201603149 NCT02728531	15	I	1st	Recruiting	03/30/2016	NA	t(11;14) and/or CCND1 overex	18–65
MDACC	Wang	RI → Hyper-CVAD	WINDOW I NCT02427620	100	II	1st	Recruiting	4/15/2015	Preliminary [46] CRR 100% mOS and mPFS NR	Blastoid or pleomorphic or, Ki-67 ≥ 30% or mutation TP53, c-MYC or NOTCH	18–65
RPCI	Hernandez-Ilizaliturri	O-HyperCVAD/O-MA	I 201611 NCT01527149	37	II	1st	Recruiting	12/12/2011	NA	t(11;14) and/or CCND1 overex and/or bcl-1/IgH rearragement	18–70
MSKCC	Zelenetz	R-CHOP14 → R-HIDAC → RIT → HDT → ASCR	11-095 NCT01484093	96	I and II	1st	Active, not rec	11/29/2011	NA	CCND1 or D2 or D3 positive	18–70

*ASCT* autologous stem cell transplant; *bcl-1* B cell leukemia/lymphoma 1; *CCND1* cyclin D1; *CHT* chemotherapy; *CRR* complete response rate; *EE* estimated enrollment; *HDT/ASCR* high-dose chemotherapy and autologous stem cell rescue; *ID* identification; *IgH* immunoglobulin heavy locus; *IHBDH* Institute of Hematology & Blood Diseases Hospital Chinese Academy of Medical Sciences & Peking Union Medical College; *MA* high-dose cytarabine and methotrexate; *mOS* median overall survival; *mPFS* median progression-free survival; *MR* maintenance rituximab; *MSKCC* Memorial Sloan Kettering Cancer Center; *MTp* maintenance thalidomide and prednisone; *MV* maintenance bortezomib; *NA* not available; *NCT* clinical trial number registered at clinicaltrials.gov; *NR* not reached; *O* ofatumumab; *overex* overexpression; *PI* principal investigator; *R* rituximab; *RAC* rituximab and cytarabine; *R-CHOP-14* rituximab, cyclophosphamide, doxorubicin, vincristine, and prednisone every 2 weeks; *rec* recruiting; *R-DHAP* rituximab cisplatin, cytosine arabinoside, and dexamethasone; *R-EDOCH* rituximab etoposide, dexamethasone, doxorubicin, cyclophosphamide, and vincristine; *R-HIDAC* rituximab and high-dose cytarabine; *RI → Hyper-CVAD* rituximab ibrutinib followed by hyperfractionated cyclophosphamide, vincristine, doxorubicin, and dexamethasone; *RIT* radioimmunotherapy Iodine ^131^I Tositumomab; *RPCI* Roswell Park Cancer Institute; *t* translocation; *UCSF* University of California, San Francisco; *V* bortezomib; *WUSM* Washington University School of Medicine

The outcomes of intensive therapies followed or not by ASCT will be illustrated through the following selected studies and clinical trials.

The intensive R-Hyper-CVAD/cytarabine/MTX has been studied in many phase II trials. In particular, the 15-year follow-up of a phase II study from the MD Anderson Cancer Center with 97 patients aged (median age 61 years) with UMCL showed that at a median follow-up of 13.4 years, the median failure-free survival (FFS) and overall survival (OS) for all patients was 4.8 and 10.7 years, respectively. However, the FFS seems to have plateaued after 10 years, with an estimated 15-year FFS of 30% in younger patients (≤ 65 years) and the 10-year cumulative incidence of MDS/acute myeloid leukemia (AML) of patients in first remission was 6.2% [10].

However, the SWOG Study S1106, a randomized phase II with 52 UMCL patients aged 33–66 years assessed R-Hyper-CVAD/cytarabine/MTX versus rituximab plus bendamustine as pre-transplant induction regimen for future development. Patients received either four cycles of R-Hyper-CVAD/cytarabine/MTX or six cycles of RB, followed by ASCT. Fifty-three of a planned 160 patients were accrued due to an unacceptably high mobilization failure rate (29%) on the R-Hyper-CVAD/cytarabine/MTX arm, which prompted premature study closure. The estimated 2-year progression-free survival (PFS) was 81 vs. 82%, and 2-year overall survival (OS) was 87 vs. 88% for BR and R-Hyper-CVAD/cytarabine/MTX, respectively. As a matter of fact, R-Hyper-CVAD/cytarabine/MTX is not an ideal platform for future multi-center transplant trials in MCL. BR achieved a 2-year PFS of 81% and a 78% MRD-negative rate. Premature closure of the study limited the sample size and the precision of PFS estimates and MRD rates [45]. This highlights that stem cell harvest should be performed early if such a strategy is contemplated. Nevertheless, prospective studies that compare intensified induction regimens have not been performed [2].

In an attempt to reduce toxicity without compromising efficacy, the single-center clinical phase II trial WINDOW I study (NCT02427620) currently open at MD Anderson Cancer Center (MDACC) treats younger UMCL patients of ≤ 65 years. The initial chemotherapy-free phase (window) consists of ibrutinib and rituximab until best response, followed by a shortened course of R-Hyper-CVAD/cytarabine/MTX in part 2. If a patient is in complete response (CR) in part 1, only four cycles of R-Hyper-CVAD/cytarabine/MTX in part 2 will be necessary. After the part 2, no ASCT or maintenance therapy will be required. The primary outcomes are ORR and the toxicity of ibrutinib and rituximab. The preliminary results presented at the ASH conference of December 2016 [46] and at the ICML (International Conference on Malignant Lymphoma) of Lugano of June 2017 [47] showed an unprecedented efficacy of the ibrutinib-rituximab combination, which represent a powerful alternative to chemotherapy. Indeed, for the 50 evaluable patients, the overall response rate (ORR) to part 1 alone (ibrutinib plus rituximab) is 100% (*n* = 50) with partial response (PR) in 20% (*n* = 10) and CR in 80% (*n* = 40). The ORR to both part 1 and part 2 (*n* = 33) was 100% and was equal to the complete response (CR) rate (100%, *n* = 33), i.e., all have achieved a CR to part 1 and part 2. After a median follow-up of 15.9 months, the median DOR (duration of response), PFS, and OS have not been reached. In part 1, the most common grade 1–2 non-hematological (non-heme) adverse effects (AEs) were fatigue (50), diarrhea (28), rash (29), myalgia (41), oral mucositis (52), peripheral neuropathy (19), nausea (25), blurred vision (19), edema (23), constipation (18), dry eyes (18), and dizziness (22). Grade 3 non-heme AEs included fatigue (4), nausea (2), infection (3), and dyspnea (2). No grade 4–5 non-heme toxicities were observed in part 1. Grade 3–4 heme AEs included lymphocytosis (22), thrombocytopenia (13), and leukopenia (15). In part 2, there was no grade 5 hematologic toxicity. The toxicity after intensive immune-chemotherapy in shortened cycles is much improved compared to historical controls, but longer follow-up is needed. In conclusions, these preliminary data indicate that the chemotherapy-free induction with ibrutinib and rituximab in newly diagnosed, young MCL patients was efficacious and well-tolerated. This unprecedented efficacy and safety may provide a window of opportunity for less chemoimmunotherapy needed for consolidation and increased survival, which will require a long-term follow-up [46]. However, this trial requires four cycles of R-Hyper-CVAD/cytarabine/MTX in part 2, which is toxic. In the future, further improvement is needed and the trial *Window II* is coming. The Window I study is currently recruiting participants and is expected to be completed in June 2021.

Ofatumumab is a fully human CD20 monoclonal antibody that targets a novel epitope on the CD20 molecule which allows closer binding to the cell surface, which is thought to contribute to both its increased ability to activate complement-dependent cytotoxicity (CDC) and the longer off-rate compared to rituximab. Ofatumumab has shown activity in the treatment of different hematological malignancies including chronic lymphocytic leukemia (CLL) [48, 49] and follicular lymphoma [50]. The only current published study using ofatumumab for the treatment of mantle cell lymphoma is a phase II trial with 12 patients with RRMCL treated with ofatumumab monotherapy. The response rate was disappointing with an ORR of only 8.3% and a median OS of 11.2 months. The trial was halted at the first predetermined evaluation point as insufficient response had been obtained [51].

Whereas, I 201611 (NCT01527149) is a phase II one-arm trial sponsored by Roswell Park Cancer Institute (two locations in the USA) using ofatumumab in the regimen O-HyperCVAD/O-MA (ofatumumab in combination with cyclophosphamide, doxorubicin hydrochloride, vincristine sulfate, and dexamethasone alternating with ofatumumab in combination with cytarabine and methotrexate) with patients aged 18–70 years with UMCL. The primary outcome is the proportion of patients experiencing a complete response, and I 201611 is currently recruiting participants with an expected primary completion date for October 2018. There are currently no preliminary results.

Alternatively, the following regimens are considerate of intermediate intensity. For instance, R-EPOCH (rituximab etoposide, prednisone, doxorubicin, cyclophosphamide, and vincristine) has been studied in the relapsed refractory setting of MCL, but very little as frontline [52]. Notably, Neelapu et al. studied this regimen in an early phase trial with 26 patients with UMCL aged 22–73 years (median age 57 years), followed by an autologous tumor-derived idiotype vaccine. This resulted in an impressive *response* with ORR of 100%, CRR of 92%, median PFS of 24 months and median OS of 104 months. With a 10-year median potential follow-up, the granulocyte-macrophage colony-stimulating factor cytokine response mediated by antitumor T cells was significantly associated with OS [53, 54].

The Institute of Hematology & Blood Diseases, Chinese Academy of Medical Sciences, and Peking Union Medical College sponsored IIT2015007-EC-1 (NCT02858804), a phase IV study for patients with UMCL aged 18–65 years, who will receive EDOCH (etoposide, dexamethasone, doxorubicin, cyclophosphamide, and vincristine) with or without rituximab alternating with DHAP with or without rituximab. If a partial remission or better response is achieved, patients will be recommended to receive autologous stem cell transplantation as consolidation therapy or another two cycles EDOCH±R/DHAP±R chemotherapy (based on patient’s choice). Patients with less than PR response will quit this study. After the end of induction and consolidation, maintenance therapy with rituximab or thalidomide plus prednisone will be given less than 2 years. The determination of maintenance regimens is dependent on patient’s choices. The primary outcome of IIT2015007-EC-1 was the PFS with a time frame up to 36 months. This study is currently recruiting participants, and the primary completion is planned for December 2019; there are no results available.

In a phase III European study of the European Mantle Cell Lymphoma Network, 455 patients aged 65 years and younger (median age 55 years) with UMCL were randomized to receive either six courses of R-CHOP followed by myeloablative radiochemotherapy and ASCT (control group) or six courses of alternating R-CHOP or R-DHAP followed by a high-dose cytarabine-containing conditioning regimen and ASCT (cytarabine group). After a median follow-up of 6.1 years, time to treatment failure was significantly longer in the cytarabine group (median 9.1 years, 5-year rate 65%) than in the control group (3.9 years, 40%). Since this pivotal trial, immunochemotherapy containing high-dose cytarabine followed by ASCT has been considered standard of care in patients aged 65 years or younger with untreated mantle cell lymphoma [55].

Alternatively, in the phase II second Nordic Mantle Cell Lymphoma trial (MCL2), 159 patients with UMCL aged 65 years and younger (median age 56 years) received alternating courses of maxi-CHOP and high-dose Ara-C followed by either BEAM or BEAC before ASCT. After a median follow-up of 11.4 years, the median overall and progression-free survival were 12.7 and 8.5 years, respectively, without reaching a plateau [9].

Another novel use of combination chemotherapy and consolidation ASCT is that of a single-center phase II study report where 23 transplant-eligible patients with UMCL aged 42–69 years (median age 57 years), of whom 70% were MCL International Prognostic Index (MIPI) low-risk, received three cycles of BR, followed by interim computer tomography (CT) restaging. Patients with progressive disease (PD) went off study; those with stable disease (SD) or better went on to receive three cycles of RC (rituximab, high-dose cytarabine). Ninety-six percent of patients achieved a CR/unconfirmed CR after treatment, and 21 patients underwent successful stem cell collection and ASCT. After a median follow-up of 13 months, the PFS rate was 96% and among 15 MRD-evaluable patients who completed treatment, 93% achieved MRD negativity after BR/RC. Therefore, BR/RC achieves very high CR and MRD negativity rates in transplant-eligible patients, with a satisfactory safety profile. RB/RC warrants further comparative studies and may become a useful alternative to R-CHOP-based induction regimens in this patient population [56].

Similarly, 201603149 (NCT02728531) is a phase I trial sponsored by the Washington University School of Medicine with bendamustine and rituximab alternating with cytarabine and rituximab for patients aged 18–65 years with UMCL. The primary objective is the stem cell mobilization success rate, and this study is currently recruiting participants with a completion date expected for September 2018.

The Memorial Sloan Kettering Cancer Center (MSKCC) sponsored 11-095 (NCT01484093), a phase I and II one-arm trial for patients with advanced stage UMCL aged 18–70 years old treated sequentially with an induction therapy of R-CHOP, a consolidation therapy of R-HIDAC (rituximab and high-dose cytarabine) followed by Iodine ^131^I Tositumomab followed by high-dose chemotherapy and autologous stem cell rescue (ASCR).

^131^I Tositumomab is monoclonal antibody-based CD20-targeted radioimmunotherapy for the treatment of CD-20-positive follicular non-Hodgkin lymphoma, with or without transformation that is refractory to rituximab and has relapsed following chemotherapy. ^131^I Tositumomab has been studied with MCL patients, but mostly in the relapsed setting [57, 58]. Interestingly, MSKCC used this agent in an early phase study followed by CHOP chemotherapy as initial therapy for UMCL patients either ineligible for or unwilling to undergo high-dose therapy and stem cell transplantation. Among the 24 patients treated (median age 66 years, range 45–80 years), at the completion of delivery therapy, the ORR was 86% and CRR 67%. With a median follow-up of 2.1 years, the 2.1-year OS was 92%. In conclusion, ^131^I Tositumomab is a very active agent in the treatment of MCL, but unfortunately, the study showed that MRD was not effectively eliminated by subsequent CHOP chemotherapy [59].

It is to be noted that as of February 2014, tositumomab and iodine ^131^I (Bexxar®) has been discontinued by the manufacturer and is no longer available [60].

The primary outcomes of 11-095 are the maximum tolerated dose (MTD) and the 3-year event-free survival (EFS). This study is ongoing but not recruiting participants, and the estimated primary completion date is expected for November 2018. However, since GlaxoSmithKline (GSK) discontinued the manufacture and sale of the BEXXAR®, it is unclear if the results of this trial will ever be published.

## Trials for patients of all ages

The following trials (Table 4) are or were open to patients 18 years and older, including patients older than 70 years, with various intensities of regimen. As previously mentioned, in this situation, the trade-off between chance of cure/toxicity and comorbidities assessment has to be carefully evaluated to allow the best chances of long-term survival.

**Table 4:** Clinical trials for patients with MCL aged 18 and older

Leading institution	PI last name	Regimen	Study ID/NCTNCT	EE	Phases	Line	Recruitment	First received	Study results	Cytogenetical inclusion criteria	Age (years)
UW	Chang	BG → risk adapted MG	UW16086 NCT03311126	32	II	1st	Recruiting	09/28/2017	NA	CCND1 positive	≥ 18
MDACC	Wang	Ibrutinib in low-risk disease	2016-0914 NCT03282396	30	II	1st	Not yet rec	09/12/2017	NA	CCND1 positive, but no RF*	≥ 18
Acerta Pharma BV	Sim-Wages	Acalabrutinib-BR	ACE-LY-106 NCT02717624	48	Ib	1st and RR	Active, not rec	02/24/2016	NA	Not specified	≥ 18
HCB	Giné	RI with indolent disease	GELTAMO-IMCL-2015 NCT02682641	50	II	1st	Recruiting	1/18/2016	NA	Indolent MCL with Ki-67 ≤ 30%	18–99
MSKCC	Kumar	1. R-R-CHOP 2. R-HIDAC 3. M R-R	15-196 NCT02633137	45	II	1st	Recruiting	12/15/2015	NA	Not specified	≥ 18
NWU	Kaplan	Intensive Induction → MI	NU 14H06 NCT02242097	36	II	1st	Recruiting	9/12/2014	NA	Not specified	≥ 18
MSKCC	Hamlin	Ofatumumab+ − bendamustine	11-050 NCT01437709	30	II	1st	Active, not rec	09/19/2011	NA	Not specified	≥ 18
PSH	Pu	VCR	NCT01439750	50	I/II	1st and relapsed	Active, not rec	8/16/2011	NA	Not specified	≥ 18
GSK	GSK	^131^I Tositumomab →CHOP	393229/005 NCT00992992	25	II	1st	Completed	10/8/2009	NA	Not specified	≥ 18
MDACC	Wang	V-R-HyperCVAD/V-R-HD-MTX/AraC	2006-0697 NCT00477412	110	I	1st	Active, not rec	05/21/2007	NA	Diffuse, nodular, blastoid	18–79
NIH	Wilson	EPOCH-R-B → MV vs observation	05-C-0170 NCT00114738	52	II	1st	Active, not rec	6/17/2005	NA	Not specified	18–100
Corixa Corporation	NA	^131^I Tositumomab →CHOP	CP-99-037 NCT00022945		II	1st	Completed	8/16/2001	NA	Not specified	≥ 18

*BR* bendamustine, rituximab; *BG* bendamustine, obinutuzumab; *CCND1* cyclin D1; *EPOCH* rituximab, etoposide, prednisone, vincristine, cyclophosphamide, and doxorubicin; *EE* estimated enrollment; *GSK* GlaxoSmithKline; *HCB* Hospital Clínic de Barcelona; *ID* identification; *MG* maintenance obinutuzumab; *M R-R* maintenance R-R; *MI* maintenance ibrutinib; *MV* bortezomib maintenance; *NA* not available; *NCT* clinical trial number registered at clinicaltrials.gov; *NIH* National Institute of Health; *NWU* Northwestern University; *PI* principal investigator; *PSH* PennState Health Milton S. Hershey Medical Center; *R-CHOP* rituximab, cyclophosphamide, doxorubicin, vincristine, and prednisone; *rec* recruiting; *R-EPOCH* rituximab, etoposide, prednisone, vincristine, cyclophosphamide, and doxorubicin EPOCH-R-B: R-EPOCH and bortezomib; RF* blastoid variant histology, pleomorphic variant histology, Ki-67 ≥ 50%, high-risk MCL International Prognostic Index (MIPI), bulky tumors > 3 cm, presence of B symptoms; *R-HIDAC* : rituximab and high-dose cytarabine; *RI* ibrutinib and rituximab; *R-R* lenalidomide and rituximab; *RR* relapsed/refractory; *VCR* bortezomib, cladribine, and rituximab; *V-R-HyperCVAD/V-R-HD-MTX/AraC* bortezomib (Velcade) plus rituximab Cyclophosphamide, Doxorubicin Hydrochloride, Vincristine Sulfate, and Dexamethasone alternating with bortezomib plus rituximab-high-dose methotrexate/cytarabine; *UW* University of Wisconsin, Madison

Most of these trials combine different therapies including novel agents and include regimen of variable intensities.

MDACC is launching in 2018 another phase II trial (2016-0914) for UMCL patients with low-risk disease (patients have been observed for 3–6 months with no progression as per imaging assessments, without the following risk factors: blastoid variant histology, pleomorphic variant histology, Ki-67 ≥ 50%, high-risk MIPI, bulky tumors > 3 cm, presence of B symptoms). Patients will be treated with ibrutinib daily until disease progression or intolerance for a maximum of 3 years. The primary outcome is PFS, and the estimated completion date is expected for 2022.

Alternatively, the Grupo Español de Linfomas y Transplante Autólogo de Médula Ósea sponsors the multi-centric Spanish phase II one-arm trial GELTAMO-IMCL-2015 (NCT02682641) with patients aged 18–99 years old with indolent UMCL (asymptomatic nodal low tumor burden (lymph node enlargement ≤ 3 cm in the maximum diameter) patients, non-nodal MCL presentation with mainly bone marrow or peripheral blood involvement, Ki-67 ≤ 30%; blastic and pleomorphic variants are excluded). In this currently recruiting trial, these patients with an indolent clinical form of MCL will be treated with rituximab and ibrutinib. The primary outcome is the rate of complete remission, and the estimated completion date of the study is for January 2020.

Northwestern University sponsors the phase II one-arm trial NH 14H06 (NCT02242097) evaluating ibrutinib maintenance following intensive induction (at least four cycles of R-CHOP with or without alternating R-DHAP with or without ASCT, or HyperCVAD with or without ASCT, or BR with or without ASCT) for patients aged 18 years and older with previously UMCL. The primary outcome is progression-free survival (PFS) rate after 2 years, and this study is currently recruiting participants with an expected completion date for June 2018.

Acerta Pharma BV sponsored the phase Ib trial ACE-LY016 (NCT02717624) with treatment-naive and relapse/refractory mantle cell lymphoma patients aged 18 years and older treated with acalabrutinib in combination with bendamustine and rituximab. The primary outcome is the number of participants with treatment-emergent adverse events, and this study is active but not recruiting participants with a completion date that is expected for February 2021.

The University of Wisconsin Carbone Cancer Center (UWCCC) is currently recruiting patients aged 18 years and older for a phase II single-arm, open-label, multi-center study (UW16086). This trial evaluates the efficacy and safety of the combination of induction chemoimmunotherapy with four to six cycles bendamustine and obinutuzumab. The treatment is followed by consolidation therapy and maintenance therapy with obinutuzumab in subjects achieving an objective response to induction therapy (i.e., complete or partial response; stable disease with objective evidence of tumor shrinkage). Subjects who are MRD-negative (determined by MRD testing on bone marrow and PB) after consolidation therapy will omit maintenance therapy. The primary outcome is the PFS at 2 years, and the trial is expected to be completed in 2024.

Alternatively, Memorial Sloan Kettering Cancer Center sponsors the phase II trial 11-050 (NCT01437709) with UMCL ineligible for ASCT with patients aged 18 years and older, treated with either single-agent ofatumumab (arm closed) or ofatumumab and bendamustine. The primary outcome is efficacy of single-agent ofatumumab or ofatumumab and bendamustine, and the study is currently ongoing but not recruiting participants with a completion date expected for September 2017.

Memorial Sloan Kettering Cancer Center sponsors the phase II one-arm trial 15-196 (NCT02633137) with patients aged 18 years and older with UMCL treated with sequential chemoimmunotherapy with lenalidomide, R-CHOP, and R-HiDAC followed by rituximab and lenalidomide maintenance. The primary outcome is the 3-year progression-free survival, and the study is currently recruiting participants with an expected completion date for December 2018.

Cladribine, (2-chlorodeoxyadenosine, 2-CdA) a purine analogue resistant to deamination by adenosine deaminase, has shown activity in different hematological malignancies and has been studied in MCL as monotherapy or combined therapy.

The Milton S. Hershey Medical Center sponsors the PSHCI 10-011 NCT01439750 phase I/II trial of bortezomib, cladribine, and rituximab (VCR) in newly diagnosed and relapsed mantle cell lymphoma (MCL) patients aged 18 years or older. This trial is active, is not recruiting, and is expected to be completed in August 2018.

Interestingly, a similar trial with VCR was recently published. This phase II open-label study with 24 patients with UMCL, RRMCL, and indolent lymphomas showed the following results: ORR 100%, CRR 50%, and 2-year PFS 82% for UMCL and RRMCL [61].

MDACC sponsored the phase I trial 2006-0697 (NCT00477412) with patients aged 18–79 years with UMCL treated with bortezomib and rituximab-HyperCVAD alternating with bortezomib plus rituximab-high-dose methotrexate/cytarabine (VR-HyperCVAD/VR-MA). The primary outcome is the maximum tolerated dose (MTD) of VR-HyperCVAD/VR-MA. This study is ongoing but not recruiting participants, and the estimated primary completion date is expected for April 2019.

The National Cancer Institute (NCI) sponsors a phase II one-arm trial 05-C-0170 (NCT00114738), open to patients aged 18–100 years with UMCL. All patients will then receive six cycles of dose-adjusted (DA)-EPOCH-BR, and if they have at least a PR, this will be followed by randomization to either immediate bortezomib maintenance × 18 months, or observation, followed by bortezomib if progression occurs. This study has, as a primary goal, to describe progression-free survival (PFS) and overall survival of early bortezomib maintenance versus observation following induction with bortezomib followed by DA-EPOCH-BR. This study is ongoing but not recruiting participants; the estimated completion date is for October 2018. As cited above, R-EPOCH has been used in the relapsed/refractory setting for MCL with ORR of 68% [52], but there are currently no published data regarding the use of R-EPOCH in the frontline setting of MCL.

Finally, ^131^I Tositumomab, as mentioned above is monoclonal antibody-based CD20-targeted radioimmunotherapy. GlaxoSmithKline sponsored the phase II trial 393229/005 (NCT00992992), and Corixa Corporation sponsored the phase II trial CP-99-037 (NCT00022945). Both trials used Iodine-131 anti-B1 antibody and CHOP for patients with previously untreated mantle cell lymphoma. GlaxoSmithKline closed 393229/005 and the CP-99-037 has not been updated on clinicaltrials.com since 06/23/2005.

As mentioned earlier, in February 2014, tositumomab and iodine ^131^I 131 (Bexxar®) has been discontinued by the manufacturer and is no longer available [60]. Consequently, it is unclear if further results will be published.

## Future directions

There has been an accrual of more and more novel therapies in the past years, in particular of immune therapies. Clinical priorities for the frontline therapy of MCL include optimizing induction therapy [62] with the aim of killing all MCL cells at the first strike with a potent intense targeted frontline therapy to eliminate any chance for a secondary resistance and to cause a long-term remission. Besides, MRD is emerging as one on the next endpoints in the management of MCL [63]. If ideally optimized, a frontline therapy could become a shortcut to cure MCL with reduced toxicity.

Moreover, increasingly sophisticated technologies brought us in the molecular area, where more molecular diversity/heterogeneity and complexity in cancer are being observed. This unveils a “mismatch” between our traditional canonical clinical trial system that selects patients based on common characteristics such as histology to evaluate a drug (drug-centric approach) and optimal treatment based on curated, individualized drug combinations for each patient (patient-centric approach). Indeed, the patient-centric approach will evaluate each patient with his/her unique set of genomic aberration [64] (such as ATM (ataxia telangiectasia mutated) [65], CCND1 (cell cycle regulatory protein cyclin D1) [66], ONC201 [67], KMT2D (histone-lysine *N*-methyltransferase 2D) [68], BET (bromodomain and extra-terminal) [69], CD200 [70]) to find common pathway themes (such as the Wnt pathway [71]) to tailor a combination of agents to the precise portfolio of abnormalities. Nevertheless, our current genomic platforms may be assessing just the tip of the iceberg of malignant complexity, which could be significantly amplified if we interrogate tumors by not only genomics but also transcriptomics, proteomics, epigenetics, and more. Consequently, this new patient-centric approach will require to remodel the way clinical research is currently done and will emphasize the importance of identifying biomarkers [72] that will enhance understanding of MCL pathology, better define biologic risk groups, and enhance prognostic ability [62, 73].

## Conclusions

Mantle cell lymphoma is a rare disease with limited resources and its treatment targeting pathways are a composition of the treatment of other hematological malignancies such as follicular lymphoma, multiple myeloma, and other solid tumors. The survival of MCL patients is improving and the therapeutic landscape is rapidly evolving and it will be imperative to incorporate genomic and molecular profiling of tumor cells into future trials [74]. Before we embrace the future, some of the presented breakthrough clinical trials show the importance frontline intensive therapy integrating novel agents and desintensified chemotherapy which target not only the tip but the underwater portion of the iceberg. However, a very potent frontline, even with less conventional cytotoxic agents, aiming for long-term cure can be a double-edged sword and the choice of therapy has to be weighted not only according to age but to a global internistic/geriatric assessment. For a more fragile population, chemotherapy-free alternatives are on the way. In the meantime, we believe that the best management of any patient with UMCL is in a clinical trial.

## Abbreviations

ABR: Acalabrutinib, rituximab, and bendamustine
ACP-196: Acalabrutinib
ADCC: Antibody-dependent cellular cytotoxicity
AE: Adverse event
AML: Acute myeloid leukemia
AraC: Cytarabine
ASCR: Autologous stem cell rescue
ASCT: Autologous stem cell transplant
ASH: American Society of Hematology
BCR: B cell receptor
BEAC: Carmustine, etoposide, ara-C, cyclophosphamide
BEAM: Carmustine, etoposide, ara-C, melphalan
BMI: Body mass index
BR: Bendamustine rituximab
BTK: Bruton’s tyrosine kinase
CAR-T: Chimeric antigen receptor (CAR)-T cells
CCND1: Cyclin D1
CDC: Complement-dependent cytotoxicity
CHOP: Cyclophosphamide, doxorubicin, vincristine, prednisolone
CLL: Chronic lymphocytic leukemia
cMCL: Classic MCL
CR: Complete response
CRR: Complete response rate
CT: Computer tomography
DOR: Duration of response
ECOG: Eastern Cooperative Oncology Group
EDOCH: Etoposide, dexamethasone, doxorubicin, cyclophosphamide, and vincristine
EFS: Event-free survival
FFS: Failure-free survival
FTRST: Flemish Version of the Triage Risk Screening Tool
IBR: Ibrutinib-BR
ICML: International Conference on Malignant Lymphoma
IGHV: Immunoglobulin heavy chain variable region
ISMCN: In situ mantle cell neoplasia
L-BR: Lenalidomide, bendamustine, and rituximab
MCL: Mantle cell lymphoma
MDACC: MD Anderson Cancer Center
MDS: Myelodysplastic syndromes
MIPI: MCL International Prognostic Index
MR: Maintenance rituximab
MRD: Minimal residual disease
MSKCC: Memorial Sloan Kettering Cancer Center
MTD: Maximum tolerated dose
mTOR: Mammalian target of rapamycin kinase
NHL: Non-Hodgkin lymphoma
NK: Natural killer
O-HyperCVAD/O-MA: Ofatumumab in combination with cyclophosphamide, doxorubicin hydrochloride, vincristine sulfate, and dexamethasone alternating with ofatumumab in combination with cytarabine and methotrexate
ORR: Overall response rate
OS: Overall survival
PD: Progressive disease
PFS: Progression-free survival
PR: Partial response
PS: Performance status
RBAC500: Rituximab, bendamustine, cytarabine 500 mg/m^2^
RBV: Rituximab, bendamustine, and bortezomib
RC: Rituximab, high-dose cytarabine
R-DHAP: Rituximab, dexamethasone, high-dose cytarabine, and cisplatin
R-EPOCH: Rituximab etoposide, prednisone, doxorubicin, cyclophosphamide, and vincristine
R-Hyper-CVAD/R-MA: Rituximab plus fractionated cyclophosphamide, vincristine, doxorubicin, and dexamethasone alternating with rituximab plus high-dose methotrexate-cytarabine
RRMCL: Relapsed/refractory MCL
SD: Stable disease
SPM: Secondary primary malignancy
UMCL: Untreated MCL
VCR: Bortezomib, cladribine and rituximab
Vcr-CAP: Bortezomib, rituximab, cyclophosphamide, doxorubicin, and prednisone
Vcr-CVAD: Bortezomib with the modified R-HyperVCAD
VR-CAP: R-CHOP regimen, but replacing vincristine with bortezomib
VR-CHOP: Bortezomib-R-CHOP
WHO: World Health Organization

## References

[1] Zhou Y, Wang H, Fang W, Romaguer JE, Zhang Y, Delasalle KB, Kwak L, Yi Q, Du XL WM (2008). Incidence trends of mantle cell lymphoma in the United States between 1992 and 2004. Cancer.

[2] Cheah CY, Seymour JF, Wang ML (2016). Mantle cell lymphoma. J Clin Oncol.

[3] Swerdlow SH, Campo E, Pileri SA, Harris NL, Stein H, Siebert R, Advani R, Ghielmini M, Salles GA. The 2016 revision of the World Health Organization classification of lymphoid neoplasms. 2016;127(20):2375–90. 10.1182/blood-2016-01-643569 PMC4874220 26980727

[4] Eskelund CW, Dahl C, Hansen JW, Westman M, Kolstad A, Pedersen LB, Montano-Almendras CP, Husby S, Freiburghaus C, Ek S (2017). TP53 mutations identify younger mantle cell lymphoma patients who do not benefit from intensive chemoimmunotherapy. Blood.

[5] Aukema SM, Hoster E, Rosenwald A, Canoni D, Delfau-Larue M-H, Rymkiewicz G, Thorns C, Hartmann S, Kluin-Nelemans H, Hermine O, et al. Expression of TP53 is associated with outcome of MCL independent of MIPI and Ki-67 in trials of the European-MCL Network. Blood. 2017. 10.1182/blood-2017-07-797019. 10.1182/blood-2017-07-797019 29196411

[6] Narurkar R, Alkayem M, Liu D (2016). SOX11 is a biomarker for cyclin D1-negative mantle cell lymphoma. Biomarker research.

[7] Martin P, Coleman M, Leonard JP (2009). Progress in mantle-cell lymphoma. J Clin Oncol.

[8] Dreyling M, Campo E, Hermine O, Jerkeman M, Le Gouill S, Rule S, Shpilberg O, Walewski J, Ladetto M (2017). Newly diagnosed and relapsed mantle cell lymphoma: ESMO Clinical Practice Guidelines for diagnosis, treatment and follow-up. Ann Oncol.

[9] Eskelund CW, Kolstad A, Jerkeman M, Raty R, Laurell A, Eloranta S, Smedby KE, Husby S, Pedersen LB, Andersen NS, et al. 15-year follow-up of the Second Nordic Mantle Cell Lymphoma trial (MCL2): prolonged remissions without survival plateau. Br J Haematol. 2016;175(3):410–8. 10.1111/bjh.14241. 10.1111/bjh.14241 27378674

[10] Chihara D, Cheah CY, Westin JR, Fayad LE, Rodriguez MA, Hagemeister FB, Pro B, McLaughlin P, Younes A, Samaniego F (2016). Rituximab plus hyper-CVAD alternating with MTX/Ara-C in patients with newly diagnosed mantle cell lymphoma: 15-year follow-up of a phase II study from the MD Anderson Cancer Center. Br J Haematol.

[11] Merli F, Luminari S, Ilariucci F, Petrini M, Visco C, Ambrosetti A, Stelitano C, Caracciolo F, Di Renzo N, Angrilli F (2012). Rituximab plus HyperCVAD alternating with high dose cytarabine and methotrexate for the initial treatment of patients with mantle cell lymphoma, a multicentre trial from Gruppo Italiano Studio Linfomi. Br J Haematol.

[12] Chen Y, Wang M, Romaguera J (2014). Current regimens and novel agents for mantle cell lymphoma. Br J Haematol.

[13] Pophali P, Rosenthal AC, Tun H, Reeder CB, Micallef IN, Porrata L, Ansell SM, Johnston P, Inwards DJ (2017). Autologous stem cell transplantation for mantle cell lymphoma in the elderly (≥65 years of age): the Mayo Clinic experience. Blood.

[14] Sawalha Y, Radivoyevitch T, Tullio K, Dean RM, Pohlman B, Hill BT, Kalaycio M, Majhail NS, Jagadeesh D (2017). The role of upfront autologous hematopoietic cell transplantation in the treatment of mantle cell lymphoma, a population based study using the National Cancer Data Base (NCDB). Blood.

[15] Soubeyran P, Gressin R (2016). Treatment of the elderly patient with mantle cell lymphoma. Hematology American Society of Hematology Education Program.

[16] Kochenderfer JN, Somerville RPT, Lu T, Shi V, Bot A, Rossi J, Xue A, Goff SL, Yang JC, Sherry RM (2017). Lymphoma remissions caused by anti-CD19 chimeric antigen receptor T cells are associated with high serum interleukin-15 levels. J Clin Oncol.

[17] Turtle CJ, Hanafi LA, Berger C, Hudecek M, Pender B, Robinson E, Hawkins R, Chaney C, Cherian S, Chen X (2016). Immunotherapy of non-Hodgkin’s lymphoma with a defined ratio of CD8+ and CD4+ CD19-specific chimeric antigen receptor-modified T cells. Sci Transl Med.

[18] Bao H, Bi C, Li W, Zhang X, Zhang H, Meng B, Fu K (2017). Chimeric antigen receptor-engineered exosome as a drug delivery system in mantle cell lymphoma. Blood.

[19] Chen W, Du X, Luo C, Zhang Q, Wang M (2016). Anti-CD19 chimeric antigen receptor T cells improve responses to chemotherapy-refractory mantle cell lymphoma: a case report. Blood.

[20] Abramson JS, Palomba L, Gordon LI, Lunning M, Arnason J, Forero-Torres A, Albertson TM, Exton VS, Sutherland C, Xie B (2016). Transcend NHL 001: immunotherapy with the CD19-directed CAR T-cell product JCAR017 results in high complete response rates in relapsed or refractory B-cell non-Hodgkin lymphoma. Blood.

[21] Obstfeld AE, Frey NV, Mansfield K, Lacey SF, June CH, Porter DL, Melenhorst JJ, Wasik MA (2017). Cytokine release syndrome associated with chimeric-antigen receptor T-cell therapy: clinicopathological insights. Blood.

[22] Martin P, Ruan J, Leonard JP (2017). The potential for chemotherapy-free strategies in mantle cell lymphoma. Blood.

[23] Dahi PB, Tamari R, Devlin SM, Maloy M, Bhatt V, Scordo M, Goldberg J, Zelenetz AD, Hamlin PA, Matasar MJ (2014). Favorable outcomes in elderly patients undergoing high-dose therapy and autologous stem cell transplantation for non-Hodgkin lymphoma. Biol Blood Marrow Transplant.

[24] Abrahamsson A, Dahle N, Jerkeman M (2011). Marked improvement of overall survival in mantle cell lymphoma: a population based study from the Swedish Lymphoma Registry. Leuk Lymphoma.

[25] Romaguera JE, Fayad L, Rodriguez MA, Broglio KR, Hagemeister FB, Pro B, McLaughlin P, Younes A, Samaniego F, Goy A (2005). High rate of durable remissions after treatment of newly diagnosed aggressive mantle-cell lymphoma with rituximab plus hyper-CVAD alternating with rituximab plus high-dose methotrexate and cytarabine. J Clin Oncol.

[26] Kahl BS, McGovern J, Blank J, Jaslowski A, Bayer G, Bottner WA, Rezazadeh H, McFarland T, Hei D, Smith J (2004). Phase II study of modified hyper-CVAD with rituximab maintenance for previously untreated mantle cell lymphoma: a Wisconsin Oncology Network Study. Blood.

[27] Chang JE, Carmichael LL, Kim K, Peterson C, Yang DT, Traynor AM, Werndli JE, Huie MS, McFarland TA, Volk M (2018). VcR-CVAD induction chemotherapy followed by maintenance rituximab produces durable remissions in mantle cell lymphoma: a Wisconsin Oncology Network Study. Clin Lymphoma Myeloma Leuk.

[28] Wang ML, Blum KA, Martin P, Goy A, Auer R, Kahl BS, Jurczak W, Advani RH, Romaguera JE, Williams ME (2015). Long-term follow-up of MCL patients treated with single-agent ibrutinib: updated safety and efficacy results. Blood.

[29] Wang ML, Rule S, Martin P, Goy A, Auer R, Kahl BS, Jurczak W, Advani RH, Romaguera JE, Williams ME (2013). Targeting BTK with ibrutinib in relapsed or refractory mantle-cell lymphoma. N Engl J Med.

[30] Maddocks K, Christian B, Jaglowski S, Flynn J, Jones JA, Porcu P, Wei L, Jenkins C, Lozanski G, Byrd JC (2015). A phase 1/1b study of rituximab, bendamustine, and ibrutinib in patients with untreated and relapsed/refractory non-Hodgkin lymphoma. Blood.

[31] Younes A, Thieblemont C, Morschhauser F, Flinn I, Friedberg JW, Amorim S, Hivert B, Westin J, Vermeulen J, Bandyopadhyay N (2014). Combination of ibrutinib with rituximab, cyclophosphamide, doxorubicin, vincristine, and prednisone (R-CHOP) for treatment-naive patients with CD20-positive B-cell non-Hodgkin lymphoma: a non-randomised, phase 1b study. Lancet Oncol.

[32] Chang BY, Francesco M, De Rooij MF, Magadala P, Steggerda SM, Huang MM, Kuil A, Herman SE, Chang S, Pals ST (2013). Egress of CD19(+)CD5(+) cells into peripheral blood following treatment with the Bruton tyrosine kinase inhibitor ibrutinib in mantle cell lymphoma patients. Blood.

[33] Wang ML, Lee H, Chuang H, Wagner-Bartak N, Hagemeister F, Westin J, Fayad L, Samaniego F, Turturro F, Oki Y (2016). Ibrutinib in combination with rituximab in relapsed or refractory mantle cell lymphoma: a single-centre, open-label, phase 2 trial. Lancet Oncol.

[34] Gribben JG, Fowler N, Morschhauser F (2015). Mechanisms of action of lenalidomide in B-cell non-Hodgkin lymphoma. J Clin Oncol.

[35] Qian Z, Zhang L, Cai Z, Sun L, Wang H, Yi Q, Wang M (2011). Lenalidomide synergizes with dexamethasone to induce growth arrest and apoptosis of mantle cell lymphoma cells in vitro and in vivo. Leuk Res.

[36] Wang M, Fayad L, Wagner-Bartak N, Zhang L, Hagemeister F, Neelapu SS, Samaniego F, McLaughlin P, Fanale M, Younes A (2012). Lenalidomide in combination with rituximab for patients with relapsed or refractory mantle-cell lymphoma: a phase 1/2 clinical trial. Lancet Oncol.

[37] Goy A, Feldman TA, Leslie LA, Panchal RP, Nyirenda T, Skarbnik AP, Yannotti K, Zenreich J, Bejot CM, Stives S (2017). Phase Ib-II study of Bruton’s tyrosine kinase (BTK) inhibitor ibrutinib, w/ lenalidomide and rituximab in relapsed/refractory mantle cell lymphoma. Blood.

[38] Rummel MJ, Niederle N, Maschmeyer G, Banat GA, von Grunhagen U, Losem C, Kofahl-Krause D, Heil G, Welslau M, Balser C (2013). Bendamustine plus rituximab versus CHOP plus rituximab as first-line treatment for patients with indolent and mantle-cell lymphomas: an open-label, multicentre, randomised, phase 3 non-inferiority trial. Lancet.

[39] Wu J, Zhang M, Liu D (2016). Acalabrutinib (ACP-196): a selective second-generation BTK inhibitor. J Hematol Oncol.

[40] Wang M, Rule S, Zinzani PL, Goy A, Casasnovas O, Smith SD, Damaj G, Doorduijn J, Lamy T, Morschhauser F, et al. Acalabrutinib in relapsed or refractory mantle cell lymphoma (ACE-LY-004): a single-arm, multicentre, phase 2 trial. Lancet. 2017. 10.1016/S0140-6736(17)33108-2. 10.1016/S0140-6736(17)33108-2 PMC7864374 29241979

[41] Albertsson-Lindblad A, Kolstad A, Laurell A, Raty R, Gronbaek K, Sundberg J, Pedersen LB, Ralfkiaer E, Karjalainen-Lindsberg ML, Sundstrom C, et al. Lenalidomide-bendamustine-rituximab in untreated mantle cell lymphoma > 65 years, the Nordic Lymphoma Group phase I+II trial NLG-MCL4. Blood. 2016.10.1182/blood-2016-03-704023.

[42] Robak T, Huang H, Jin J, Zhu J, Liu T, Samoilova O, Pylypenko H, Verhoef G, Siritanaratkul N, Osmanov E (2015). Bortezomib-based therapy for newly diagnosed mantle-cell lymphoma. N Engl J Med.

[43] Till BG, Li H, Bernstein SH, Fisher RI, Burack WR, Rimsza LM, Floyd JD, DaSilva MA, Moore DF, Pozdnyakova O (2016). Phase II trial of R-CHOP plus bortezomib induction therapy followed by bortezomib maintenance for newly diagnosed mantle cell lymphoma: SWOG S0601. Br J Haematol.

[44] Campo E, Rule S (2015). Mantle cell lymphoma: evolving management strategies. Blood.

[45] Chen RW, Li H, Bernstein SH, Kahwash S, Rimsza LM, Forman SJ, Constine L, Shea TC, Cashen AF, Blum KA (2017). RB but not R-HCVAD is a feasible induction regimen prior to auto-HCT in frontline MCL: results of SWOG Study S1106. Br J Haematol.

[46] Wang M, Lee HJ, Thirumurthi S, Chuang HH, Hagemeister FB, Westin JR, Fayad LE, Samaniego F, Turturro F, Chen W (2016). Chemotherapy-free induction with ibrutinib-rituximab followed by shortened cycles of chemo-immunotherapy consolidation in young, newly diagnosed mantle cell lymphoma patients: a phase II clinical trial. Blood.

[47] Wang ML, Lee, H., Thirumurthi, S., Chuang, H et al: ICML Lugano 2017 abstract. Ibrutinib-rituximab followed by reduced chemo-immunotherapy consolidation in young, newly diagnosed mantle cell lymphoma patients: a window of opportunity to reduce chemo. Hematol Oncol*,* 35*(*S2*):* 142*–*143.

[48] Wierda WG, Padmanabhan S, Chan GW, Gupta IV, Lisby S, Osterborg A (2011). Ofatumumab is active in patients with fludarabine-refractory CLL irrespective of prior rituximab: results from the phase 2 international study. Blood.

[49] Tedeschi A, Rossi D, Motta M, Quaresmini G, Rossi M, Coscia M, Anastasia A, Rossini F, Cortelezzi A, Nador G (2015). A phase II multi-center trial of pentostatin plus cyclophosphamide with ofatumumab in older previously untreated chronic lymphocytic leukemia patients. Haematologica.

[50] Czuczman MS, Hess G, Gadeberg OV, Pedersen LM, Goldstein N, Gupta I, Jewell RC, Lin TS, Lisby S, Strange C (2012). Chemoimmunotherapy with ofatumumab in combination with CHOP in previously untreated follicular lymphoma. Br J Haematol.

[51] Furtado M, Dyer MJ, Johnson R, Berrow M, Rule S (2014). Ofatumumab monotherapy in relapsed/refractory mantle cell lymphoma—a phase II trial. Br J Haematol.

[52] Jermann M, Jost LM, Taverna C, Jacky E, Honegger HP, Betticher DC, Egli F, Kroner T, Stahel RA (2004). Rituximab-EPOCH, an effective salvage therapy for relapsed, refractory or transformed B-cell lymphomas: results of a phase II study. Ann Oncol.

[53] Neelapu SS, Kwak LW, Kobrin CB, Reynolds CW, Janik JE, Dunleavy K, White T, Harvey L, Pennington R, Stetler-Stevenson M (2005). Vaccine-induced tumor-specific immunity despite severe B-cell depletion in mantle cell lymphoma. Nat Med.

[54] Grant C, Neelapu SS, Kwak LW, Dunleavy K, White T, Miller BW, Jaffe ES, Steinberg SM, Bird BH, Wilson WH (2011). Eleven-year follow-up of idiotype vaccine and DA-EPOCH-rituximab in untreated mantle cell lymphoma: correlation of survival with idiotype immune response. Blood.

[55] Hermine O, Hoster E, Walewski J, Bosly A, Stilgenbauer S, Thieblemont C, Szymczyk M, Bouabdallah R, Kneba M, Hallek M (2016). Addition of high-dose cytarabine to immunochemotherapy before autologous stem-cell transplantation in patients aged 65 years or younger with mantle cell lymphoma (MCL Younger): a randomised, open-label, phase 3 trial of the European Mantle Cell Lymphoma Network. Lancet.

[56] Armand P, Redd R, Bsat J, Mayuram S, Giardino A, Fisher DC, AS LC, Jacobson C, Davids MS, Brown JR (2016). A phase 2 study of Rituximab-Bendamustine and Rituximab-Cytarabine for transplant-eligible patients with mantle cell lymphoma. Br J Haematol.

[57] Gopal AK, Rajendran JG, Petersdorf SH, Maloney DG, Eary JF, Wood BL, Gooley TA, Bush SA, Durack LD, Martin PJ (2002). High-dose chemo-radioimmunotherapy with autologous stem cell support for relapsed mantle cell lymphoma. Blood.

[58] Kruger PC, Cooney JP, Turner JH (2012). Iodine-131 rituximab radioimmunotherapy with BEAM conditioning and autologous stem cell transplant salvage therapy for relapsed/refractory aggressive non-Hodgkin lymphoma. Cancer Biother Radiopharm.

[59] Zelenetz AD, Noy A, Pandit-Taskar N, Scordo M, Rijo I, Zhou Y, O’Donoghue JA, Divgi C (2006). Sequential radioimmunotherapy with tositumomab/iodine I131 tositumomab followed by CHOP for mantle cell lymphoma demonstrates RIT can induce molecular remissions. J Clin Oncol.

[60] http://ca.gsk.com/en-ca/media/press-releases/2013/sk-to-discontinue-manufacture-and-sale-of-the-bexxar-therapeutic-regimen-tositumomab-and-iodine-i-131-tositumomab/ access date 23 February 2017 GtdmasotB.

[61] Puvvada SD, Guillen-Rodriguez J, Kumar A, Inclan L, Heard K, Rivera XI, Anwer F, Schatz JH, Mahadevan D, Persky DO (2018). Phase 2 open-label study of bortezomib, cladribine, and rituximab in advanced, newly diagnosed, and relapsed/refractory mantle-cell and indolent lymphomas. Clin Lymphoma Myeloma Leuk.

[62] Spurgeon SE, Till BG, Martin P, Goy AH, Dreyling MP, Gopal AK, Leblanc M, Leonard JP, Friedberg JW, Baizer L, et al. Recommendations for clinical trial development in mantle cell lymphoma. J Natl Cancer Inst. 2017;109(1) 10.1093/jnci/djw263 PMC6059122 28040733

[63] Goy A (2016). Mantle cell lymphoma: is it time for a new treatment paradigm?. Hematol Oncol Clin North Am.

[64] Ahmed M, Zhang L, Nomie K, Lam L, Wang M. Gene mutations and actionable genetic lesions in mantle cell lymphoma. Oncotarget. 2016;7(36):58638–48. 10.18632/oncotarget.10716. 10.18632/oncotarget.10716 PMC5295458 27449094

[65] Ahmed M, Li L, Pinnix C, Dabaja B, Nomie K, Lam L, Wang M (2016). ATM mutation and radiosensitivity: an opportunity in the therapy of mantle cell lymphoma. Crit Rev Oncol Hematol.

[66] Mohanty A, Sandoval N, Das M, Pillai R, Chen L, Chen RW, Amin HM, Wang M, Marcucci G, Weisenburger DD (2016). CCND1 mutations increase protein stability and promote ibrutinib resistance in mantle cell lymphoma. Oncotarget.

[67] Ishizawa J, Kojima K, Chachad D, Ruvolo P, Ruvolo V, Jacamo RO, Borthakur G, Mu H, Zeng Z, Tabe Y (2016). ATF4 induction through an atypical integrated stress response to ONC201 triggers p53-independent apoptosis in hematological malignancies. Sci Signal.

[68] **ICML** 2017 Abstract, Ferrero et al, KMT2D AND TP53 mutations predict poor pfs and os in mantle cell lymphoma receiving high-dose therapy and asct: the Fondazione Italiana Linfomi (FIL) MCL0208 phase III trial. Hematol Oncol, 35(S2): 94–95.

[69] Sun B, Shah B, Fiskus W, Qi J, Rajapakshe K, Coarfa C, Li L, Devaraj SG, Sharma S, Zhang L (2015). Synergistic activity of BET protein antagonist-based combinations in mantle cell lymphoma cells sensitive or resistant to ibrutinib. Blood.

[70] Hu Z, Sun Y, Schlette EJ, Tang G, Li S, Xu J, Yin CC, Young KH, Patel KP, Miranda RN, et al. CD200 expression in mantle cell lymphoma identifies a unique subgroup of patients with frequent IGHV mutations, absence of SOX11 expression, and an indolent clinical course. Mod Pathol. 2017. 10.1038/modpathol.2017.135. 10.1038/modpathol.2017.135 28984300

[71] Mathur R, Sehgal L, Braun FK, Berkova Z, Romaguerra J, Wang M, Rodriguez MA, Fayad L, Neelapu SS, Samaniego F (2015). Targeting Wnt pathway in mantle cell lymphoma-initiating cells. J Hematol Oncol.

[72] Inamdar AA, Goy A, Ayoub NM, Attia C, Oton L, Taruvai V, Costales M, Lin YT, Pecora A, Suh KS. Mantle cell lymphoma in the era of precision medicine-diagnosis, biomarkers and therapeutic agents. Oncotarget. 2016. 10.18632/oncotarget.8961 PMC5217048 27119356

[73] Wheler J, Lee JJ, Kurzrock R (2014). Unique molecular landscapes in cancer: implications for individualized, curated drug combinations. Cancer Res.

[74] Kumar A (2015). Novel agents in mantle cell lymphoma. Curr Oncol Rep.

